# Electrochemical Study on Newly Synthesized Chlorocurcumin as an Inhibitor for Mild Steel Corrosion in Hydrochloric Acid

**DOI:** 10.3390/ma6125466

**Published:** 2013-11-27

**Authors:** Ahmed A. Al-Amiery, Abdul Amir H. Kadhum, Abu Bakar Mohamad, Ahmed Y. Musa, Cheong Jiun Li

**Affiliations:** 1 Department of Chemical and Process Engineering, University of Kebangsaan Malaysia (UKM), Bangi, Selangor 43000, Malaysia; E-Mails: amie@eng.ukm.my (A.A.H.K.); drab@eng.ukm.my (A.B.M.); jiunli@gmail.com (C.J.L.); 2 Environmental Research Center, University of Technology (UOT), Baghdad 10001, Iraq; 3 Department of Chemistry, Western University, 1151 Richmond Street, London, Ontario N6A3K7, Canada; E-Mail: amusa6@uwo.ca

**Keywords:** corrosion inhibition, chlorocurcumin, polarization, potentiodynamic

## Abstract

A new curcumin derivative, *i.e.*, (1E,4Z,6E)-5-chloro-1,7-bis(4-hydroxy-3-methoxyphenyl)hepta-1,4,6-trien-3-one (chlorocurcumin), was prepared starting with the natural compound curcumin. The newly synthesized compound was characterized by elemental analysis and spectral studies (IR, ^1^H-NMR and ^13^C-NMR). The corrosion inhibition of mild steel in 1 M HCl by chlorocurcumin has been studied using potentiodynamic polarization (PDP) measurements and electrochemical impedance spectroscopy (EIS). The inhibition efficiency increases with the concentration of the inhibitor but decreases with increases in temperature. The potentiodynamic polarization reveals that chlorocurcumin is a mixed-type inhibitor. The kinetic parameters for mild steel corrosion were determined and discussed.

## 1. Introduction

**Figure 1:**
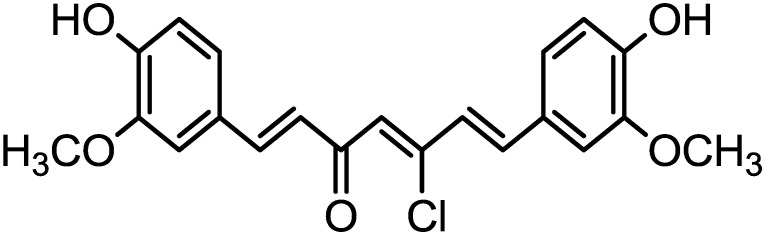
Structure of chlorocurcumin.

Natural Products (NPs) have traditionally played an important role in industrial applications, including the discovery of drug NPs, which were the basis for most early medicines [1,2]. Many organic compounds have been studied to investigate their corrosion inhibition potential, e.g., the effect of organic nitrogen compounds on the corrosion behavior of iron and steel in acidic solutions; these organic nitrogen compounds are usually employed for their rapid action [3,4]. The effective and efficient corrosion inhibitors are those compounds that have π-bonds, contain hetero-atoms such as sulfur, nitrogen, oxygen and phosphorous and allow the adsorption of compounds on the metal surface [5,6,7,8,9]. The organic inhibitors decrease the corrosion rate by adsorbing on the metal surface and blocking the active sites by displacing water molecules, leading to the formation of a compact barrier film on the metal surface. Most organic inhibitors are toxic, highly expensive and environmentally unfriendly. Thermodynamic adsorption parameters and kinetic corrosion parameters have been utilized to describe inhibitor adsorption behavior. Riggs and Hurd [10] reported that the heat of inhibitor adsorption could be obtained by comparing the activation energies of uninhibited and inhibited corrosion reactions. However, while a positive heat of adsorption, differential heat of adsorption (DHads.) DHads. > 0 (endothermic process), has been unequivocally attributed to chemisorption, [10,11] a negative heat of adsorption, DHads. < 0 (exothermic process), could involve physisorption, [12] chemisorption [13,14] or a mixture of both processes (comprehensive adsorption) [14,15,16,17,18,19]. It is well established that the effect of temperature on the inhibition of an acid–metal reaction is highly complex, due to the many changes that occur on the metal surface, such as the rapid etching and desorption of the inhibitor, and because the inhibitor itself may undergo decomposition and/or rearrangement. It was observed that few inhibitors in acid-metal systems have specific reactions that are effective at high temperatures rather than at low temperatures [15]. The temperature dependence of the inhibiting effect and a comparison of the values of the apparent activation energy, Ea, of the corrosion process in the absence and presence of the inhibitor of interest could provide further evidence concerning its mechanism of inhibition [16]. In the present study, corrosion inhibitor (1E,4Z,6E)-5-chloro-1,7-bis(4-hydroxy-3-methoxyphenyl)hepta-1,4,6-trien-3-one (chlorocurcumin) was synthesized and investigated as an inhibitor for the corrosion of mild steel in 1.0 M hydrochloric acid (HCl) using electrochemical impedance spectroscopy (EIS), potentiodynamic polarization (PDP) and electrochemical frequency modulation (EFM). The structure for the synthesized novel corrosion inhibitor proposed based on spectroscopic evidence and is shown in Figure 1.

## 2. Results and Discussion

### 2.1. Polarization Measurements

**Figure 2:**
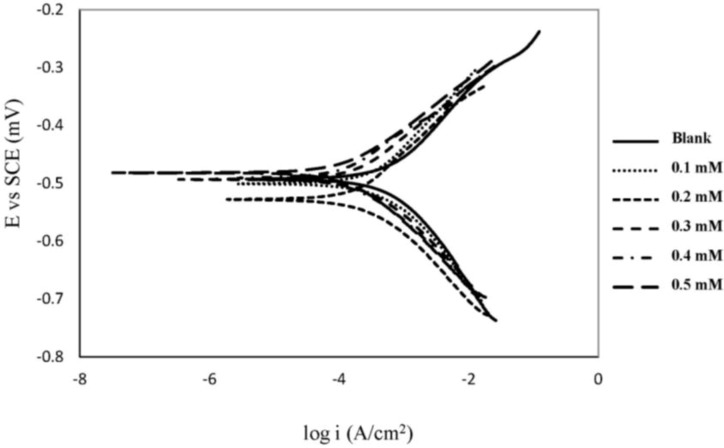
Potentiodynamic polarization curves for mild steel in 1.0 M HCl at 30 °C with different concentrations of chlorocurcumin.

The polarization curves for mild steel in 1.0 M HCl solutions at various concentrations of chlorocurcumin at 30 °C are shown in Figure 2. The values for corrosion current density (*i*_corr_), corrosion potential (*E*_corr_), anodic Tafel slope (β_a_), cathodic Tafel slope (β_c_) and inhibition efficiency (*IE*%) are displayed in Table 1. These values were calculated from the Tafel fit routine provided by the Gamry Echem analyst software, which uses a non-linear chi-squared minimization to fit the data to the Stern-Geary equation. The inhibition efficiency was calculated as follows:

(1) $$IE(\%)=\frac{i_{corr}-i_{corr(inh)}}{i_{corr}}\times 100$$

where *i*_corr_ and *i*_corr(inh)_ are the corrosion current densities without and with the addition of inhibitor, respectively.

**Table 1:** Polarization parameters for mild steel in 1.0 M HCl with different concentrations of chlorocurcumin at 30 °C.

Concentration (mM)	β_a_ (V dec^−1^)	β_c_ (V dec^−1^)	*i*_corr_ (μA cm^−2^)	−*E*_corr_ (mV *vs.* SCE)	*IE*%
Blank	0.12	0.14	660.1	491	0.00
0.1	0.15	0.13	421.0	501	36.22
0.2	0.12	0.11	254	528	61.52
0.3	0.10	0.11	232	493	64.85
0.4	0.09	0.10	150	491	77.28
0.5	0.09	0.11	145	482	78.03

A compound can be classified as an anodic- or cathodic-type inhibitor when the displacement in *E*_corr_ is larger than 85 mV with respect to *E*^0^_corr_ [20,21]. Because the largest displacement of *E*_corr_ occurs in the presence of chlorocurcumin, then the molecules can be considered a mixed-type inhibitor. Adding chlorocurcumin to HCl solution reduces the anodic dissolution of mild steel and retards the cathodic hydrogen evolution.

Table 1 shows that the *i*_corr_ values decreased in the presence of chlorocurcumin. This result indicates that the corrosion rate decreases with the addition of inhibitor and that the inhibition efficiency increases with inhibitor concentration. The slight change in the values of the Tafel constants (β_a_, β_c_) with the addition of chlorocurcumin indicates that the inhibitor controlled both reactions. This result also indicates that the adsorbed molecules did not affect the mechanism of mild steel dissolution or hydrogen evolution [22].

### 2.2. Electrochemical Impedance Spectroscopy (EIS) Measurements 

**Figure 3:**
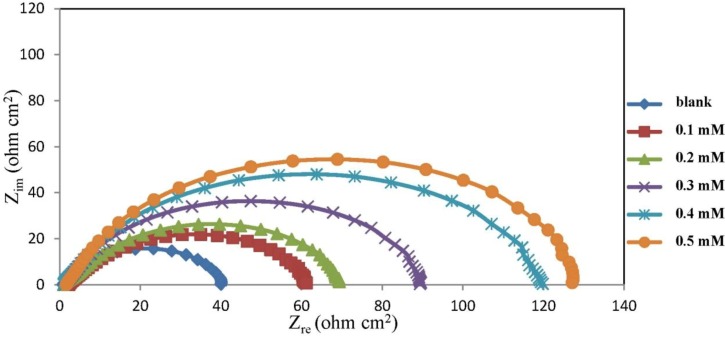
Nyquist plot for mild steel in 1.0 M HCl at 30 °C with different concentrations of chlorocurcumin.

The experimental results obtained from EIS measurements for the corrosion of mild steel in the presence and absence of chlorocurcumin at 30 °C are listed in Table 2. The Nyquist plot for mild steel in the absence and presence of various concentration of chlorocurcumin is shown in Figure 3. The increase in the diameter of the semicircle indicates that impedance increases with increasing inhibitor concentration. In general, the impedance spectra exhibit one single depressed semicircle, and the diameter of the semicircle increases with the increase of inhibitor concentration. The semicircle can be attributed to the charge transfer that takes place at electrode/solution interface, and the transfer process controls the corrosion reaction of mild steel. The existence of a single semicircle shows the single charge transfer process during dissolution which is unaffected by the presence of inhibitor molecules [23,24]. The semicircle-shaped Nyquist plots imply that the formation of a barrier on the surface and the corrosion of mild steel are largely controlled by a charge transfer process [25]. The existence of single semicircle showed the single charge transfer process during dissolution which is unaffected by the presence of inhibitor molecules. Deviation of perfect circular shape is often referred to the frequency dispersion of interfacial impedance. This anomalous behavior is generally attributed to the inhomogeneity of the metal surface arising from surface roughness or interfacial phenomena [26,27]. The obtained semicircles cut the real axis at higher and lower frequencies. The intercept corresponds to *R*_s_ at the higher frequency (Table 2) end and to *R*_s_ + *R*_t_ at the lower frequency end, where *R*_t_ is the difference between these two values [28].

**Table 2:** Impedance parameters for mild steel in 1.0 M HCl with different concentrations of chlorocurcumin at 30 °C.

Concentration (mM)	*R*_s_ (ohm cm^−2^)	*R*_ct_ (ohm cm^−2^)	CPE_dl_ Y_o_X10^−5^ (Ss^α^/cm^2^)	α	*C*_dl_ (μF cm^−2^)	*IE*%
0	–	42.00	–	0.78	294.47	0.00
0.1	2.15	61.45	60.48	0.74	190.21	31.65
0.2	0.69	87.85	47.60	0.76	174.68	52.19
0.3	1.12	91.55	38.86	0.81	177.69	54.12
0.4	0.94	121.40	27.37	0.82	159.64	65.40
0.5	1.65	130.80	29.10	0.84	126.14	67.89

Note: *R_s_*, solution resistance; R_ct_, charge-transfer resistance; CPE, constant phase element; α denotes an empirical parameter.

**Figure 4:**
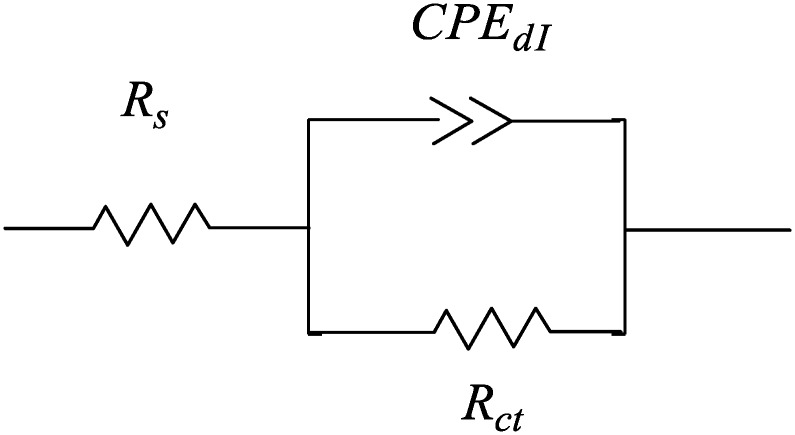
Equivalent circuit model used to fit the impedance data for mild steel.

The EIS spectra were analyzed using the equivalent circuit in Figure 4. The constant phase element (CPE) is represented as follows:

(2) $$Z(\omega )=Y_{o}.{\left(j\omega \right)}^{-\alpha }$$

where *Y*_o_ is the CPE constant, ω is the angular frequency (rad/s), *j*^2^ = −1 and is an imaginary number and α is the CPE exponent. The double layer charge transfer, *C*_dl_, can be calculated as follows:

(3) $$C_{dl}={\left(Y_{o}R_{ct}^{1-\alpha }\right)}^{1/\alpha }$$

To confirm the polarization results, the inhibition efficiencies (*IE*%) for mild steel in 1.0 M HCl is calculated as follows:

(4) $$IE(\%)=\frac{R_{ct}-R_{ct}^{o}}{R_{ct}}\times 100$$

where *R*_ct_ and *R*^0^_ct_ are the charge transfer resistances with and without inhibitor, respectively.

The *IE*% increased with inhibitor concentration, and this result was in agreement with those obtained from the potentiodynamic polarization measurements. It can be observed form Table 2 that the presence of chlorocurcumin increases the values of *R*_ct_ and reduces the *C*_dl_ values. The increased *R*_ct_ values are due to the formation of a protective film on the metal surface that prevents mass and charge transfer [29]. Conversely, the decrease in *C*_dl_ is attributed to the increase in the film layer thickness formed by the adsorption of inhibitor molecules [30,31,32].

### 2.3. Corrosion Kinetic Parameters

The effect of temperature on the corrosion parameter of mild steel in 1.0 M HCl was studied at 30, 40, 50 and 60 °C. The activation energy (*E*_a_), activation enthalpy (∆*H*_a_) and activation entropy (∆*S*_a_) for the corrosion of mild steel in 1.0 M HCl with and without the inhibitor were determined using the Arrhenius and transition state plots.

The activation energy can be obtained from the Arrhenius equation and plot:

(5) $$i_{\text{corr}}=A\text{exp}\left(-\frac{E_{\text{a}}}{RT}\right)$$

where *i*_corr_ is the corrosion current (A·cm^−2^), *A* is pre-exponential factor, *E*_a_ is activation energy (J·mol^−1^), *R* is gas constant (8.314 J·mol^−1^·K^−1^) and *T* is temperature in K.

Taking the logarithm yields:

(6) $$\text{ln }i_{\text{corr}}=\left(\frac{-E_{\text{a}}}{R}\right)\left(\frac{1}{T}\right)+\text{ln }A$$

**Figure 5:**
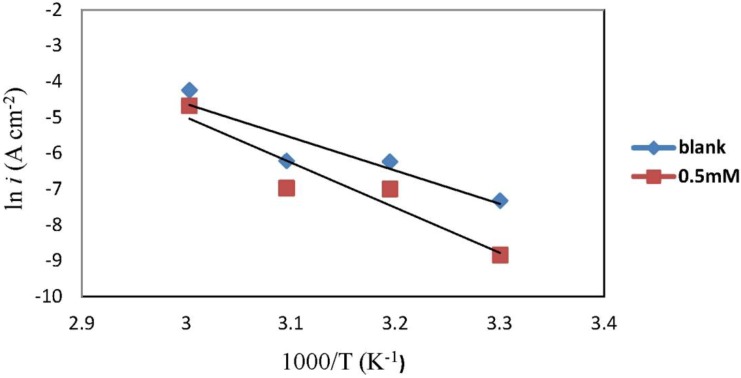
Arrhenius plot for mild steel in 1.0 M HCl.

The graph of ln (*i*_corr_) against 1000/*T* yields a straight line with the slope equal to (*−E*_a_/*R*). The Arrhenius plot for mild steel in 1.0 M HCl in the presence and absence of chlorocurcumin is shown in Figure 5. The values of *E*_a_ were calculated and are displayed in Table 3.

**Table 3:** Corrosion kinetic parameters for mild steel in 1.0 M HCl in the presence and absence of chlorocurcumin.

Concentration	*E*_a_ (kJ·mol^−1^)	∆*H*_a_ (kJ·mol^−1^)	∆*S*_a_ (J·mol^−1^·K^−1^)
Blank	77.25	74.61	−60.44
0.5 mM	104.74	102.10	18.91

The transition state equation was used to calculate the ∆*H*_a_ and ∆*S*_a_:

(7) $$i_{\text{corr}}=\frac{RT}{hN}\text{exp}\left(\frac{\Delta S_{a}}{R}\right)\text{exp}\left(\frac{-\Delta H_{a}}{RT}\right)$$

where *N* is Avogadro’s number (6.02 × 10^23^ mol^−1^) and *h* is Plank’s constant (6.63 × 10^−34^ m^2^·kg·s^−1^).

To carry out simple calculations, Equation (7) was rearranged to become

(8) $$\text{ln }\left(\frac{i_{\text{corr}}}{T}\right)=\left(\frac{-\Delta H_{a}}{R}\right)\left(\frac{1}{T}\right)+\left[\text{ln }\left(\frac{R}{Nh}\right)+\frac{\Delta S_{a}}{R}\right]$$

**Figure 6:**
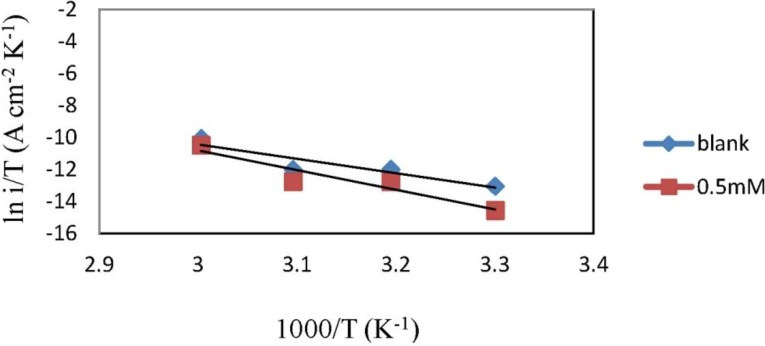
Transition state plots for mild steel in 1.0 M HCl.

A plot of ln (*i*_corr_/*T*) against 1000/*T* gives a straight line with the slope equal to (−∆*H*_a_/*R*) and intercept equal to [ln(*R*/*Nh*) + (∆*S*_a_/*R*)], as shown in Figure 6. The ∆*H*_a_ and ∆*S*_a_ values were calculated and are displayed in Table 3.

From Table 3, the activation energy increases in the presence of the inhibitor, implying that a physical adsorption (electrostatic) process occurred in the initial stage. In addition, the *E*_a_ values are greater than 20 kJ·mol^−1^ in both the presence and absence of the inhibitor, which indicate that the entire process is controlled by the surface reaction [33]. According to Szauer and Brand, the increase in activation energy can be attributed to the decrease in the adsorption of the inhibitor on the mild steel surface with increases in temperature [34]. The values of *E*_a_ and ∆*H*_a_ are higher in the presence of the inhibitor. This result shows that the energy barrier of the corrosion reaction is increased without changing the mechanism of dissolution. The endothermic nature of steel dissolution is indicated by the positive values of ∆*H*_a_ for both the corrosion processes with and without the inhibitor.

Meanwhile, the positive values of ∆*S*_a_ reveals that the adsorption process is accompanied by a increase in the entropy which acts as a driving force for adsorption of the inhibitor on the mild steel surface. The value of ∆*S*_a_ increases in the presence of the inhibitor and is generally interpreted by increases in the disorder, as the reactants are converted to activated complexes [35].

### 2.4. Adsorption Isotherm 

The adsorption isotherm was collected to investigate the interaction between the inhibitor and the mild steel surface. The surface coverage values (θ) were obtained from the polarization measurements and are calculated thus:

(9) $$\theta =\frac{i_{\text{corr}}^{\text{o}}-i_{\text{corr}}}{i_{\text{corr}}^{\text{o}}}$$

**Figure 7:**
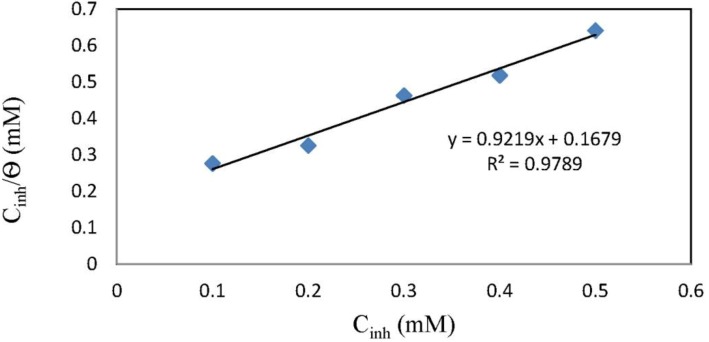
Adsorption isotherm for chlorocurcumin on the mild steel surface in 1.0 M HCl.

It was assumed that the adsorption of chlorocurcumin follows the Langmiur adsorption isotherm model. Plotting *C*_inh_/θ against *C*_inh_ (Figure 7) resulted in a straight line, which indicates that the inhibitor adsorption obeys the Langmiur adsorption isotherm. The Langmiur adsorption isotherm is represented by Equation (10):

(10) $$\frac{C_{\text{inh}}}{\theta }=\frac{1}{K_{\text{ads}}}+C_{\text{inh}}$$

where *C*_inh_ is the concentration of the inhibitor and *K*_ads_ is the adsorption constant that is related to the standard free energy of adsorption, ∆*G*^0^_ads_ as follow:

∆*G*^0^_ads_ = −*RT* ln(55.5*K*_ads_) (11)

Generally, values of ∆*G*^0^_ads_ up to −20 kJ/mol are consistent with physisorption, while those approximately −40 kJ/mol or lower associated with chemisorption. Chemisorption involves charge sharing or charge transfer between the metal and organic molecules [36,37]. In this case, the calculated value for ∆*G*^0^_ads_ is −14.61 kJ/mol; the mechanism of adsorption for chlorocurcumin on mild steel is thus physisorption.

## 3. Experimental Section

All chemical**s** used were of reagent grade (supplied by either Malaysia|Sigma-Aldrich) and used as received without further purifications. The Fourier transform infrared (FTIR) spectra were measured using a Thermo Scientific Model Nicolate 6700 Spectrophotometer. NMR spectra were recorded on a Model AVANCE III 600 MHz spectrometer.

### 3.1. Synthesis of Chlorocurcumin

Curcumin (1.23 g, 0.00334 mol) was mixed with POCl_3_ (10 mL). The resulting suspension was refluxed for approximately 3 hours, then cooled to room temperature and slowly poured onto crushed ice mixed with water. The violet solid formed was collected by filtration, washed with ice-water and recrystallized from acetonitrile. For the FTIR spectrum of chlorocurcumin, the broad peak at 3412.9 cm^−1^ was due to O–H stretching vibration while the sharp peak at 1727.8 was due to carbonyl stretching. Both peaks at 2959.7 cm^−1^ and 2931.6 cm^−1^ were assigned to aromatic rings on chlorocurcumin. Peaks at 1598.8 cm^−1^ and 1513.0 cm^−1^ are the typical C=C stretch vibration. ^1^H NMR (CDCl_3_): δ (ppm) 7.5 (H–C=C); 7.27 (H–aromatic); 4.2 (–OH); 0.9 (–OCH_3_). ^13^C NMR (CDCl_3_): δ (ppm) 167.7 (C=O); 128-132 (C-aromatic); 68.18 (C–Cl); 38.8(–OCH_3_) [38].

### 3.2. Electrochemical Measurements

Mild steel specimens obtained from Metal Samples Company were used as the working electrodes throughout the study. The composition (wt %) of the mild steel was as follows: Fe, 99.21; C, 0.21; Si, 0.38; P, 0.09; S, 0.05; Mn, 0.05; Al, 0.01. The active surface area of the mild steel was 4.5 cm^2^. The specimens were cleaned according to ASTM standard procedure G1-03 [39,40,41,42]. The measurements were carried out in aerated, non-stirred 1.0 M hydrochloric acid solutions at 30, 40, 50 and 60 °C at a concentration range of 0.1–0.5 mM chlorocurcumin as the corrosion inhibitor. The selection of the acid concentration was based on the conditions commonly observed during the selection process at industrial facilities. Solutions were freshly prepared using distilled water. Each measurement was repeated three times, and only the average values were reported to verify the reproducibility of the experiments. The inhibitory effects of chlorocurcumin were investigated with a Gamry water jacketed glass cell, which contained three electrodes: the working, counter and reference electrode (the reference electrode consisted of a saturated calomel electrode (SCE)). The measurements were performed using a Gamry Instrument Potentiostat/Galvanostat/ZRA model REF 600.

## 4. Conclusions 

Chlorocurcumin inhibits the corrosion of mild steel in 1.0 M HCl solution. The inhibition efficiency increases with the concentration of the inhibitor. Potentiodynamic polarization studies implied that the chlorocurcumin is a mixed-type inhibitor. The activation energy showed that the entire process is controlled by the polarization resistance, surface reaction and that the adsorption of chlorocurcumin on the metal surface obeyed the Langmuir adsorption isotherm.
